# Association between Objectively Measured Physical Activity and Arterial Stiffness in Children with Congenital Heart Disease

**DOI:** 10.3390/jcm10153266

**Published:** 2021-07-24

**Authors:** Laura Willinger, Leon Brudy, Renate Oberhoffer-Fritz, Peter Ewert, Jan Müller

**Affiliations:** 1 Department of Pediatric Cardiology and Congenital Heart Disease, Deutsches Herzzentrum München, Technische Universität München, 80636 München, Germany; leon.brudy@tum.de (L.B.); renate.oberhoffer@tum.de (R.O.-F.); peter.ewert@dhm.mhn.de (P.E.); j.mueller@tum.de (J.M.); 2 Institute of Preventive Pediatrics, Technische Universität München, 80992 München, Germany

**Keywords:** physical activity, arterial stiffness, children with congenital heart disease

## Abstract

Background: The association between physical activity (PA) and arterial stiffness is particularly important in children with congenital heart disease (CHD) who are at risk for arterial stiffening. The aim of this study was to examine the association between objectively measured PA and arterial stiffness in children and adolescents with CHD. Methods: In 387 children and adolescents with various CHD (12.2 ± 3.3 years; 162 girls) moderate-to-vigorous PA (MVPA) was assessed with the “Garmin vivofit jr.” for 7 consecutive days. Arterial stiffness parameters including pulse wave velocity (PWV) and central systolic blood pressure (cSBP) were non-invasively assessed by oscillometric measurement via Mobil-O-Graph^®^. Results: MVPA was not associated with PWV (ß = −0.025, *p* = 0.446) and cSBP (ß = −0.020, *p* = 0.552) in children with CHD after adjusting for age, sex, BMI z-score, peripheral systolic blood pressure, heart rate and hypertensive agents. Children with CHD were remarkably active with 80% of the study population reaching the WHO recommendation of average 60 min of MVPA per day. Arterial stiffness did not differ between low-active and high-active CHD group after adjusting for age, sex, BMI z-score, peripheral systolic blood pressure, heart rate and hypertensive agents (PWV: F = 0.530, *p* = 0.467; cSBP: F = 0.843, *p* = 0.359). Conclusion: In this active cohort, no association between PA and arterial stiffness was found. Longer exposure to the respective risk factors of physical inactivity might be necessary to determine an impact of PA on the vascular system.

## 1. Introduction

Children with congenital heart disease (CHD) are at high cardiovascular risk as a consequence of their congenital condition. Hence, risk stratification and the identification of modifiable risk factors is particularly important [1]. Pulse wave velocity (PWV) and central systolic blood pressure (cSBP) are surrogate measures of arterial stiffness [2] and strong predictors for cardiovascular and all-cause morbidity and mortality [3,4,5]. Measures of arterial stiffness were shown to be increased already in children with CHD, predisposing this patient cohort to premature heart failure [6,7]. Physical activity (PA) is a clinically important modifiable risk factor for preventing arterial stiffening of the vessels and for determining risk of cardiovascular disease in children with CHD. The importance of PA in children and adolescents with CHD has been emphasized frequently in the recent years [8,9], yet the actual level of PA in children with CHD remains uncertain as the reported PA levels vary considerably between different studies [10,11,12,13].

In healthy children, the interplay of arterial stiffness and PA has provided contradictory findings [14,15,16,17,18,19]. Studies investigating the effect of PA on arterial stiffness in children and adolescents with CHD are rare. One study reported of increased arterial stiffness in low-active children with CHD compared to high-active children with CHD and healthy controls [20]. Another study reported of an inverse association between high levels of MVPA and lower aortic PWV in children with CHD [21]. This association is particularly important in the way of preventing cardiovascular disease in this patient cohort who showed reduced elasticity of the vascular system already in childhood [6,7]. Therefore, this study aimed to analyze the association between objectively measured PA and arterial stiffness in a large cohort of children and adolescents with CHD.

## 2. Materials and Methods

### 2.1. Study Participants

In a cross-sectional design, objectively measured PA and surrogates of arterial stiffness were assessed in 387 children and adolescents with various CHD (12.2 ± 3.3 years; 162 girls). Study participants were recruited during their routine outpatient visit at the German Heart Center Munich from March 2016 to January 2021. Study participants were free of acute infections or any neurologic diseases and without restriction to sports and exercise. Various CHDs were categorized into five major subgroups. Detailed information on the characteristics of the study population is given in Table 1. Part of the data have already been published in a cross-sectional comparison of PA levels with healthy controls [10].

### 2.2. Assessment of Arterial Stiffness

Aortic PWV and cSBP was obtained by a single measurement recording brachial oscillometric blood pressure waves using an automated Mobil-o-Graph^®^ device (I.E.M, Stolberg, Germany) as previously described [22,23]. HMS software version 4.7 with C1 calibration was used. The Mobil-o-Graph^®^ device is non-invasive and has shown good validity and reliability in several studies and is regularly used in the pediatric population [24,25,26,27]. PWV and cSBP were calculated by a proprietary algorithm of the device [28,29]. Measurements were performed at the left upper arm in a supine position after 5 minutes of rest with cuff size adjusted for individual arm circumference

### 2.3. Objective Assessment of Physical Activity

PA was objectively assessed with the “Garmin vivofit jr.” device (Garmin Ltd., Olathe, KS, USA), which children wore on their wrist for 7 consecutive days. The “Garmin vivofit jr.” is a wearable that tracks every single minute of moderate-to-vigorous PA (MVPA) and steps throughout the day. The physical activity wearable was shown to be accurate is assessing MVPA and step count [30,31].Participants and their guardians were asked to transfer the data from the device to a report sheet at the end of each day. Overall, 329 children (85%) provided complete and valid data for objective PA on 7 consecutive days. Data of at least 3 weekdays and 1 weekend day were considered the minimum to calculate a weekly average and were available for another 58 children (15%). According to the WHO guidelines on PA and sedentary behavior published 2020, children and adolescents should do at least an average of 60 min per day of MVPA across the week [32].

### 2.4. Data Analysis

Descriptive data of the children with CHD are shown in mean values, standard deviations (mean ± SD) and total numbers (%) if appropriate. MVPA and steps were analyzed for every single day and computed to weekly averages for statistical purposes. The association between PA, in the form of MVPA and step count, and surrogates of arterial stiffness was analyzed using multivariate linear regression analyses adjusted for age, sex, z-score of the body mass index (BMI), peripheral systolic blood pressure (pSBP) heart rate and intake of hypertensive agents (yes/no) as in our previous analysis on this topic [22,23]. The activity level of CHD subgroups was compared to the WHO recommendation of average 60 min per day with one-sample *t*-test. The study population was categorized as high-active and low-active based on whether children met the WHO recommendations [32]. In addition, 10,000 steps on a daily average were identified as a cut-off for low-active and high-active CHD-groups. Differences in anthropometric data were analyzed with chi-square test and *t*-test for unpaired samples. General linear models adjusted for age, sex, z-score BMI, pSBP, heart rate and intake of hypertensive agents were calculated to detect differences between the high-active and low-active CHD group in PWV and cSBP. All analyses were performed with RStudio (version 1.3.1093) and SPSS (V25.0, IBM Corporation) with the level of significance set to two-sided *p*-values < 0.050 for all tests.

## 3. Results

Daily MVPA was not associated with PWV (ß = −0.025, *p* = 0.446) and cSBP (ß = −0.020, *p* = 0.552) after adjusting for age, sex, BMI z-score, pSBP, heart rate and hypertensive agents in 387 children with CHD, (Table 2). This multivariate model explained 74.8% of the variance of PWV and of cSBP. There were no associations between daily step count and surrogates of arterial stiffness. Separate analysis for patients with and without antihypertensive treatment can be found in Supplementary Table S1.

**Figure 1:**
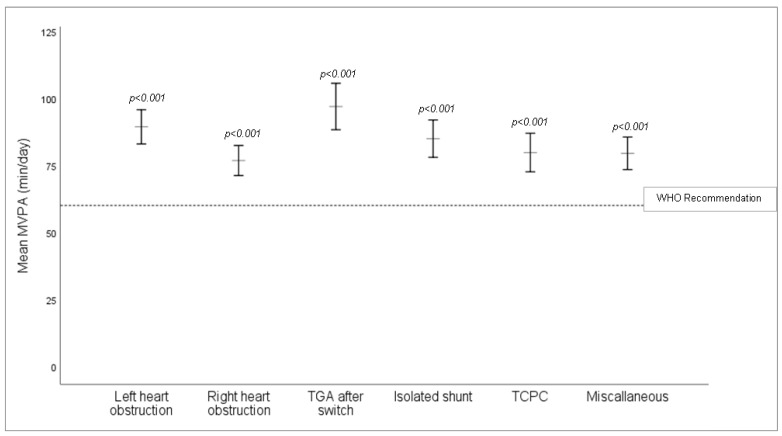
Mean values of MVPA (min/day) between CHD subgroups corrected for age, sex and BMI z-score. All CHD subgroups significantly exceeded the minimum 60 min MVPA per day recommended by the WHO. TCPC = total cavopulmonary connection, TGA = transposition of the great arteries.

Overall, children with CHD were remarkably active with a weekly average of 83.3 ± 28.1 min MVPA per day. In total, 80% of the study population (309 participants) accumulated at least 60 min of MVPA per day on a weekly average and thus met the WHO recommendations [32], (Table 1 and Figure 1). Physical activity level did not significantly differ in the CHD severity groups (*p* = 0.313, Supplementary Table S2).

PWV z-Scores based on age, body height and sex were calculated according to Elemenhorst et al. 2015 [28], with an overall PWV z-score of 0.098 ± 1.76 m/s in our study population.

Comparing the high-active and low-active group, both groups did not differ in regard to surrogates of arterial stiffness after adjusting for age, sex, BMI z-score, pSBP, heart rate and hypertensive agents for PWV (high-active: 4.55 ± 0.4 m/s vs. low-active: 4.70 ± 0.4 m/s, F = 0.53, *p* = 0.467) and cSBP (high-active: 98.9 ± 11.0 mmHg vs. low-active: 101.1 ± 9.9 mmHg, F = 0.843, *p* = 0.359), (Table 3). The results remain unchanged when evaluating 10,000 steps on a daily average as a cut-off for low-active and high-active CHD-groups.

## 4. Discussion

The main finding of the present study is that objectively measured PA was not related to arterial stiffness measures in a largely active cohort of children with CHD.

In healthy children, the relationship between PA and arterial stiffness remains unclear [14], as only half of the included studies in a recently published review reported of improved vascular function in physically active healthy children. In particular, the type of vascular measurement and the type of PA assessment has a considerable influence on reported outcomes [19].

Compared to healthy children, several diagnostic subgroups of patients with CHD show increased arterial stiffness already in the childhood [6,7]. Arterial stiffening in children with CHD has anatomic, histologic, and surgical reasons. Underlying factors are great arterial medial abnormalities of smooth muscle, elastic fibers, collagen and ground substance, impairing the natural buffering function of the vessels in children with CHD [33]. Additionally, surgical scars and resulting fibrotic tissue, implanted patches or conduits as well as pharmacological treatment foster arterial stiffness [2,34]. Due to the pre-existing pathology in children with CHD, the influence of PA becomes particularly important and sufficient evidence is lacking.

In contrast to our results, a study by Boyes and colleagues [20] with a rather small sample size of 17 children reported of increased arterial stiffness in low-active children with CHD compared to high-active children with CHD based on objectively measured daily step-counts. However, this study did not take confounders into account even though several influencing factors on arterial stiffness are well known [3], especially age, obesity and hypertension [35]. When examining the association between PA and arterial stiffness it is therefore important to consider these confounders, as otherwise the impact of PA on arterial stiffness will be biased. Furthermore, our larger sample size might contribute to a clearer picture on the association.

Lopez and colleagues [21] reported of an inverse association between MVPA and aortic PWV after adjusting for sex, age and BMI in 104 children with CHD at a mean age of 12 years. In contrast to our study their patient cohort was rather inactive with an average of 46.7 ± 20.0 min MVPA per day and only 25% of the cohort meeting the WHO recommendation, which markedly differs from the 80% in our patient cohort [21]. In our specialized tertiary center children are encouraged to be physically active during their follow-up appointments which may result in our cohort being more active than the average CHD population. Previous studies investigating PA levels in children with CHD reported rather conflicting results, as some studies showed reduced PA [11,12]. Other studies found high PA levels in children with CHD [10] with no significant differences to healthy peers [10,13]. Overall, the disease-awareness of our patients and their families who attend the regular follow-up appointments is considerably high. In combination with the high activity level, this might have been the most explanatory factor for our results. However, these findings cannot be generalized as our findings largely depend on a large high-active and small low-active CHD group. Further studies are needed to explore and understand the association of arterial stiffness and PA in high-active and low-active children with CHD.

### Limitation

As the exposure to risk factors is relatively short in studies with children it is often difficult to show correlations. In addition, when sampling patients from 8 to 18 years of age the time period is just 10 years. This narrow time window and the lack of exposure indicates the necessity for a huge sample size or a huge effect to yield significant results.

As our institution is a specialized tertiary center, complex CHD might be overrepresented in this study population. Combined with the already mention encouragement of PA in our institution the novelty and excitement of wearing the “Garmin vivofit jr.” device might have additionally increased the PA level during the measurement period [36]. Therefore, PA levels in our study cohort might be over-represented compared to reality. However, compared to recent research on PA level of children with CHD in our institution, the measured PA level seems rather normal [10].

A prior study has shown a systematic underestimation of the PWV in high PWV values with the Mobil-O-Graph^®^ device [29], which might have influenced our results even in this young cohort. The algorithm used by the Mobil-O-Graph^®^ yields only estimates of the PWV, whereby estimates of PWV were shown to be strongly dependent on age and systolic BP and less on the waveform characteristics [29,37]. Mobil-O-Graph^®^ is nevertheless considered as a good and valid method for estimating PWV as it is easy to perform, operator-independent and reliable [29]. In children and adolescents, oscillometric noninvasive estimations using Mobil-O-Graph^®^ were shown to be effective for evaluating cSBP in this age group [27].

## 5. Conclusions

No association between PA and arterial stiffness was found in this active cohort of children with CHD. The influence of PA on arterial stiffness in low-active children and patients with a longer exposure to respective risk factors needs further investigation. Nevertheless, PA promotion is indicated in patients with CHD, as its benefits are unquestionable.

## Figures and Tables

**Table 1:** Population characteristic of children with congenital heart disease.

Subgroup CHD	n	Sex ♀	Age (Years)	BMI z-Score	PWV (m/s)	cSBP (mmHg)	MVPA (min/Day)	Steps/Day
Total	387	162 (42)	12.2 ± 3.3	−0.32 ± 1.19	4.60 ± 0.44	99.3 ± 10.8	83.3 ± 28.1	9386 ± 3090
**CHD Diagnosis**								
Left heart obstruction	69	21 (30)	12.3 ± 3.3	−0.58 ± 1.18	4.55 ± 0.46	101 ± 13.8	89.2 ± 29.8	9976 ± 3387
Right heart obstruction	89	35 (39)	12.1 ± 3.0	−0.44 ± 1.43	4.58 ± 0.42	98.1 ± 10.1	77.2 ± 29.1	9233 ± 3084
Isolated shunts	57	31 (54)	12.3 ± 3.5	−0.02 ± 1.05	4.58 ± 0.42	99.8 ± 11.1	83.8 ± 26.0	9309 ± 2905
TGA after switch	37	9 (24)	11.8 ± 3.1	−0.12 ± 0.97	4.60 ± 0.38	98.7 ± 10.9	98.3 ± 29.7	11,260 ± 3460
TCPC	57	19 (33)	12.9 ± 3.5	−0.18 ± 1.03	4.66 ± 0.47	100 ± 10.4	98.3 ± 29.7	9043 ± 2768
Miscellaneous	78	47 (60)	11.9 ± 3.6	−0.39 ± 1.17	4.55 ± 0.32	98.4 ± 7.9	79.2 ± 23.4	8456 ± 2560
**Surgical status**								
Native CHD	112	51 (46)	11.8 ± 3.4	−0.26 ± 1.09	4.49 ± 0.37	98.2 ± 10.8	83.1 ± 28.8	9383 ± 3061
Heart surgery	275	111 (40)	12.4 ± 3.3	−0.35 ± 1.23	4.62 ± 0.42	99.8 ± 10.8	83.3 ± 27.8	9387 ± 3107
**CHD severity** *								
Simple	74	36 (49)	12.3 ± 3.6	−0.07 ± 1.12	4.57 ± 0.41	99.7 ± 11.1	85.4 ± 26.9	9653 ± 3034
Moderate	119	46 (39)	11.7 ± 3.1	−0.39 ± 1.16	4.57 ± 0.47	100.3 ± 13.1	81.7 ± 28.6	9365 ± 3161
Complex	171	65 (38)	12.4 ± 3.3	−0.42 ± 1.26	4.61 ± 0.38	98.7 ± 9.1	84.8 ± 28.7	9551 ± 3083
**Yes**	58	24 (41)	12.1 ± 3.4	−0.45 ± 1.09	4.41 ± 0.34	96.4 ± 11.7	74.1 ± 23.3	8336 ± 3240
**No**	329	138 (42)	12.2 ± 3.3	−0.30 ± 1.21	4.61 ± 0.41	99.8 ± 10.6	84.9 ± 28.5	9571 ± 3030

* 23 patients could not be classified by the ACC criteria. Values are number (%) or mean ± SD. BMI = body mass index, CHD = congenital heart disease, cSBP = central systolic blood pressure, MVPA = moderate to vigorous physical activity, PWV = pulse wave velocity, TGA = transposition of the great arteries, TCPC = total cavopulmonary connection.

**Table 2:** Association between surrogates of arterial stiffness and MVPA adjusted for age, sex, BMI z-score, peripheral systolic blood pressure, heart rate and hypertensive agents.

	Adjusted R^2^	ß	95%CI	*p*-Value
PWV (m/s)	0.748	−0.025	[−0.001; 0.001]	0.446
cSBP (mmHg)	0.748	−0.020	[−0.035; 0.019]	0.552

cSBP = central systolic blood pressure, MVPA = moderate to vigorous physical activity, PWV = pulse wave velocity.

**Table 3:** Population characteristic of high-active and low-active children with congenital heart disease.

	Low-Active CHD (n = 78)	High-Active CHD (n = 309)	*p*-Value
Sex, female	40 (51%)	122 (40%)	0.079
Age, years	13.3 ± 3.3	11.9 ± 3.3	0.002 †
BMI, z-score	−0.20 ± 1.6	−0.36 ± 1.1	0.298 †
MVPA, min/day	47.8 ± 11.8	92.1 ± 23.6	**<0.001** †
Steps/day	6150 ± 2014	10,203 ± 2762	**<0.001** †
PWV, m/s	4.70 ± 0.4	4.55 ± 0.4	0.467 ‡
cSBP, mmHg	101.1 ± 9.9	98.9 ± 11.0	0.359 ‡

Values are number (%) or mean ± SD. †: Student’s *t*-test for independent sample. ‡: General linear model adjusted for age, sex, BMI z-score, peripheral systolic blood pressure, heart rate and hypertensive agents. BMI = body mass index, CHD = congenital heart disease, cSBP = central systolic blood pressure, MVPA = moderate to vigorous physical activity, PWV = pulse wave velocity.

## Supplementary Materials

The following tables are available online at https://www.mdpi.com/article/10.3390/jcm10153266/s1, Table S1: Association between surrogates of arterial stiffness and MVPA adjusted for age, sex, BMI z-score, peripheral systolic blood pressure and heart rate, separated for patients treated with antihypertensive drugs and untreated patients; Table S2: Physical activity level based on MVPA of different CHD severities after adjusting for age, sex and BMI z-score.
