# Supplementary material for: Association between Objectively Measured Physical Activity and Arterial Stiffness in Children with Congenital Heart Disease
Source: J Clin Med. 2021 Jul 24;10(15):3266. doi: 10.3390/jcm10153266 (PMC8348234; doi:10.3390/jcm10153266)
Supplement: Supplementary file 1 [file jcm-10-03266-s001.zip › jcm-1264748-supplementary.pdf]

Supplementary

Table S1.: Association between surrogates of arterial stiffness and MVPA adjusted for age, sex, BMI z-score, peripheral systolic blood pressure and heart rate, separated for patients treated with antihypertensive drugs and untreated patients.

	**Adjusted R^2**	**ß**	**95%CI**	**p-value**
		**UNTREATED**		
PWV (m/s)	0.735	-0.180	[-0.001; 0.001]	0.611
cSBP (mmHg)	0.747	0.002	[-0.027; 0.028]	0.959
		**TREATED**		
PWV (m/s)	0.828	-0.060	[-0.003; 0.001]	0.427
cSBP (mmHg)	0.748	-0.128	[-0.160; 0.027]	0.158

cSBP=central systolic blood pressure, MVPA= moderate to vigorous physical activity, PWV = pulse wave velocity

Table S2.: Physical activity level based on MVPA of different CHD severities after adjusting for age, sex and BMI z-score.

**CHD Severity**	**Estimated marginal means MVPA (min/day)**	**Standard error**	**95%-CI**
Complex CHD	84.5	2.1	[80.4 – 88.7]
Moderate CHD	80.7	2.5	[75.8 – 85.6]
Simple CHD	86.7	3.2	[80.4 – 93.0]

Corrected for age, sex and BMI z-score. CHD = congenital heart disease, CI = confidence interval, MVPA= moderate to vigorous physical activity
